# Hypertranscription: the invisible hand in cancer epigenetics

**DOI:** 10.1186/s13072-026-00702-0

**Published:** 2026-09-18

**Authors:** Steven Henikoff, Robert N. Eisenman, Kami Ahmad

**Affiliations:** 1 Fred Hutchinson Cancer Center, Seattle, WA 98109 USA; 2 Howard Hughes Medical Institute, Chevy Chase, MD 20815 USA

## Abstract

Global upregulation of transcription, or hypertranscription, is observed in most human cancers, driven by the action of oncogene-encoded transcription factors (TFs). TF interactions with RNA Polymerase II (Pol II) are mediated by kinases that phosphorylate the Pol II C-terminal domain (CTD) to drive productive elongation. The combinatorial complexity of hundreds of transcription factors and kinases acting on thousands of genes that differ in expression between cell types obscures common features that may result from hypertranscription. One of these features is histone overexpression, which can accelerate cell-cycle progression and cause transcription-replication conflicts, triggering chromosomal instability. Pol II profiling provides a direct measure of hypertranscription and histone gene upregulation in cancer, a pan-cancer diagnostic to stratify patient populations and follow the course of treatment. As many broadly effective anticancer drugs target Pol II activity, a better understanding of how they counteract hypertranscription has implications for drug development and for improved treatment regimens. Here we review the underlying mechanisms that drive hypertranscription and its impact on chromosome integrity, aging and neoplasia.

## Introduction

It is by now widely accepted that cancer is both a genetic and an epigenetic disease [1]. This duality was first articulated by David Hansemann, who in 1890 described both aneuploidy, a genetic hallmark of cancer cells, and “anaplasia”, a less differentiated state, possessing a relatively greater ability to exist independently and proliferate than “altruistic” normal cells [2, 3]. Cancer genetics, including aneuploidy and mutations in oncogenes and tumor suppressor genes, has been central to the understanding of cancer biology in the modern era [4]. However, our understanding of cancer epigenetics [5] has progressed only incrementally over the past 20 years, where insights from studies of DNA methylation, histone modifications and histone variants reveal an increasingly complex picture of the anaplastic state [6, 7]. As drugs targeting these mediators of epigenetic information are widely used in treating cancer [8, 9], it is critical to understand the mechanisms whereby cancer cells enter an anaplastic state.

**Fig. 1:**
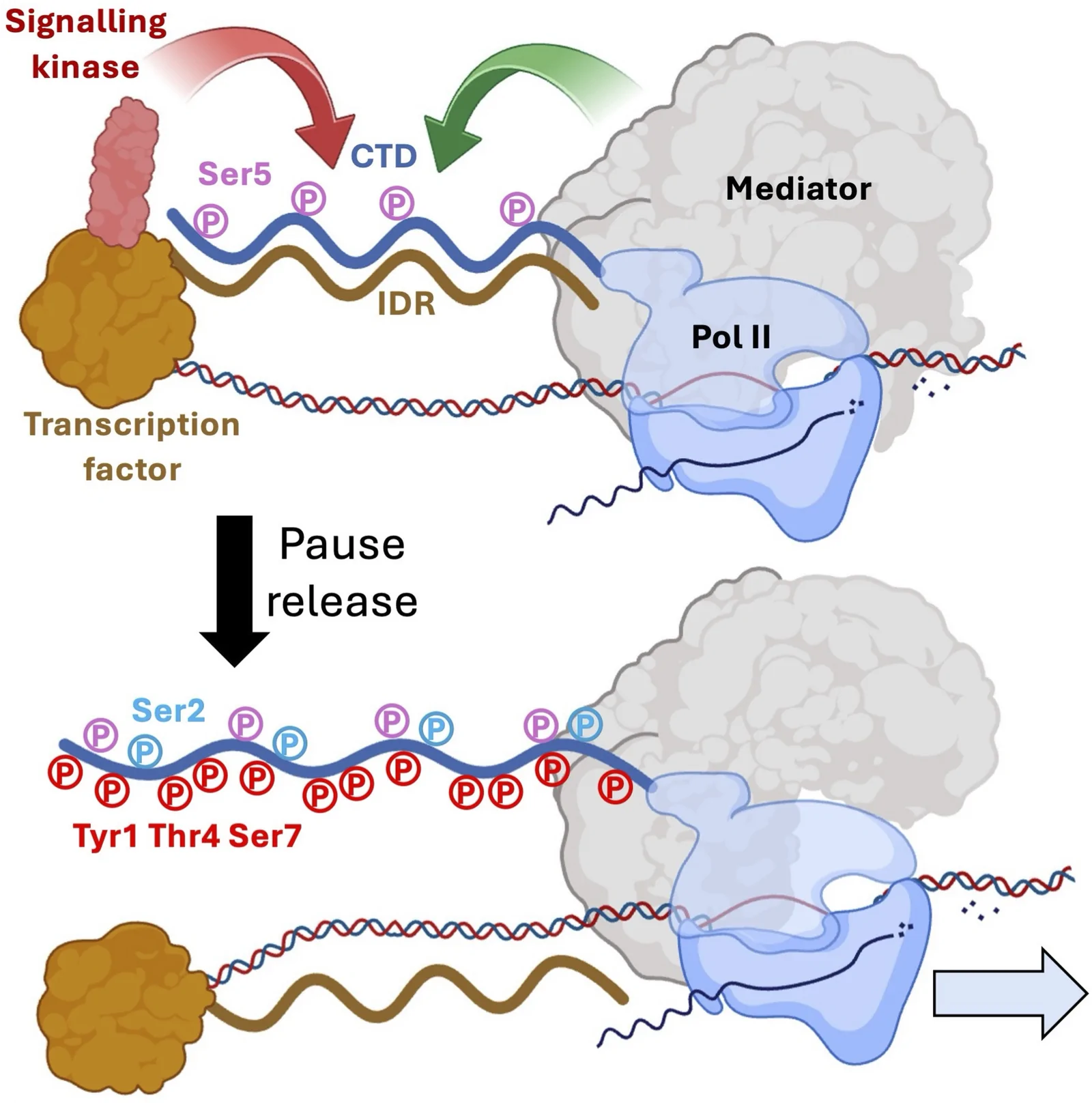
Model for RNA Polymerase II (Pol II) pause release by C-Terminal Domain (CTD) phosphorylation (Adapted from Ref [152]). Top: Engaged Pol II (blue) pauses just downstream of the promoter, becoming Serine−5 phosphorylated (circled Ps) over the tandem 52 copies of the Tyr1-Ser2-Pro3-Thr4-Ser5-Pro6-Ser7 heptapeptide that comprises the Rpb1 CTD. The CTD weakly interacts with the intrinsically disordered domain (IDR) of a nearby sequence-specific transcription factor (TF, brown) [147]. Pause release for productive elongation is triggered by accumulation of negative charges distributed over the CTD. Bottom: The kinase module of the Mediator Complex (grey) phosphorylates Ser-2 (blue circled Ps) while a signalling kinase phosphorylates Tyrosine−1, Threonine−4 or Serine−7 (red circled Ps) depending on its substrate preference. Each phosphate contributes ~2 negative charges to the CTD at physiological pH resulting in charge repulsion that extends the CTD and together with the bulk of the additional phosphates (red circled Ps from the receptor kinase and blue circled Ps from transcriptional CDKs) releases Pol II from the TF IDR. Created in BioRender. Henikoff, S. (2025) https://BioRender.com/v439hm8

Studies of cells in culture, organoids and animal models have convincingly demonstrated that cancer begins with a single cell [10], and a cancer-like state can be induced by engineered alterations of gene expression even in the absence of genetic change [11]. However, with ~30 trillion cells in an average human and with cancer cells arising daily, determining what happened in the first cell that gave rise to a tumor is unfeasible. Furthermore, solid tumors are detected only when there are hundreds of millions of cells, by which time a tumor is too heterogeneous to clearly discriminate cause from effect. Fortunately, recent studies have provided insights into the central role of RNA Polymerase II (Pol II) at the S-phase-dependent histone genes in driving the anaplastic state. Transcription factors can recruit kinases to specific DNA sequences permitting targeting of the 52 tandem copies of the C-terminal domain (CTD) of the Rpb1 core catalytic subunit, releasing Pol II from the Mediator complex [12, 13] (Fig. 1). Upregulation of oncogenic transcription factors promotes increased CTD phosphorylation and transcriptional bursting [14], resisted by nucleosomes, which are stabilized by repressive histone modifications, DNA methylation and tumor suppressor proteins [6]. Recent studies have also shown that hypertranscription, or proportional upregulation of gene expression, is a common feature of cancer cells regardless of type or stage [15–17]. Here we examine evidence that the anaplastic state is a consequence of hypertranscription driving histone overexpression, allowing more rapid progression through S-phase of the cell cycle and triggering chromosomal instability.

### Hypertranscription is common across cancer types

**Fig. 2:**
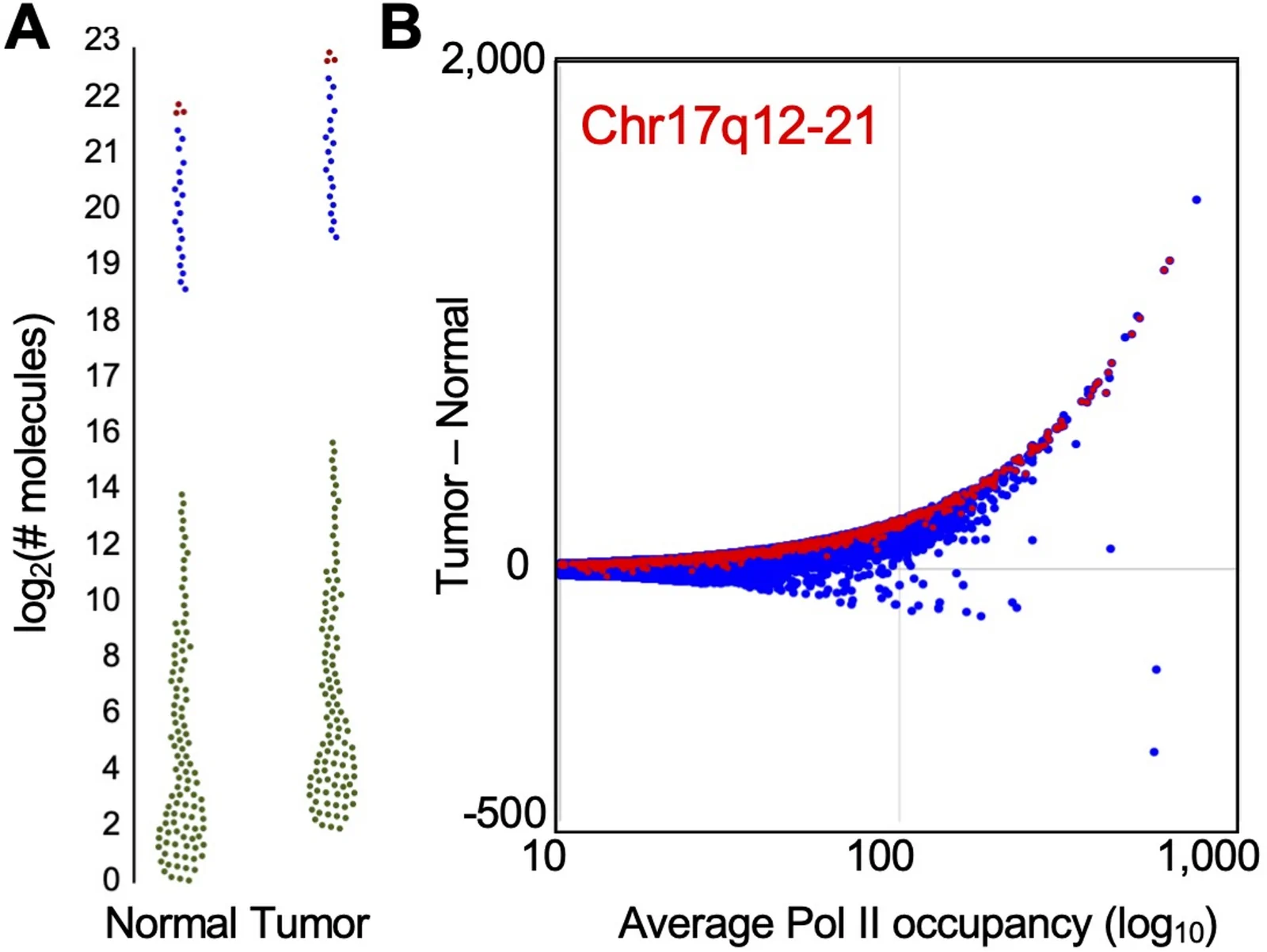
Detection of hypertranscription and focal amplifications in epigenomic data. **A** Transcript counts from individual genes (dots) span a wide range, with ~10,000 different genes transcribed by RNA polymerase II (Pol II, green dots, including protein-coding mRNA and non-coding transcripts) accounting for ~5% of all RNA molecules in a cell. The roughly 10% of total RNAs produced by RNA polymerase III (Pol III, blue dots, including tRNAs and snoRNAs) are more abundant, while the bulk (~ 85%) are ribosomal RNAs produced by RNA polymerase I (Pol I, red dots). Appropriate normalization of read counts per cell detects global hypertranscription in cancers, where abundance of all transcripts increases and may support more rapid cell growth and proliferation. An example of cancer hypertranscription is the discovery that MYC mis-regulation induces a two-fold increase in elongating Pol II genome-wide [24] and in Pol I-produced transcripts [153]. **B** Hypertranscription can be detected between a matched Tumor and Normal sample pair from a breast cancer patient using a modified Bland–Altman plot [146]. Each dot represents Pol II-Ser5p CUTAC signal for one of ~1 million single-copy candidate *cis*-Regulatory Elements (cCREs), where the difference between Tumor and Normal samples is displayed on the Y-axis. The X-axis shows the average Pol II, plotted on a log scale for clarity [33]. Red dots represent cCREs within the Chr17q12-21 region that includes the HER2 locus, a regional amplification in this tumor

Hypertranscription is genome-wide upregulation of all three eukaryotic RNA Polymerases, where RNA Polymerase I (Pol I) transcribes ribosomal DNA repeats to produce the cell’s 18S, 5.8S and 28S rRNAs for packaging into ribosomes within the nucleolus [15]. Pol II produces all of a cell’s mRNAs and most other types of RNAs, and Pol III produces tRNAs, 5S rRNA and various other small RNAs (Fig. 2A). At steady-state, rRNAs and tRNAs account for the large majority of the transcriptional output of a cell, but standard RNA-seq protocols are designed for steady-state oligo(dT)-primed mRNA transcribed by Pol II. As the polyadenylated “transcriptome” comprises only a few percent of the total RNA, it is not ideally suited for detecting hypertranscription. Instead, hypertranscription can be measured by comparing total RNA levels between populations of cells normalized by cell number using “spike-in” RNAs or genomic DNA, or by comparing total RNA levels between populations of single cells. Such assays have led to the discovery of hypertranscription in embryonic stem cells, driven by certain chromatin regulators, such as the CHD1 ATP-dependent nucleosome remodeler and the transcription factor (TF) MYC [18–20].

Hypertranscription is not the only process that produces more RNA overall while preserving the normal relative stoichiometry of transcripts. Endoreplication, in which chromosomes duplicate without mitosis, characterizes the terminally differentiated cells of insect larvae [21], and polyploidy, in which mitosis occurs without cell division, is especially common in plants and in cancer [22]. In mammals, syncytiotrophoblasts and endoreplicated cells (trophoblast giant cells) populating the placenta are examples in which normal single cells produce excess RNAs in equimolar amounts. In such cases, the RNA/DNA ratio remains the same while the RNA/cell ratio increases stepwise. By contrast, in hypertranscription, a fractional increase in the RNA/cell ratio can be accomplished without requiring a round of DNA synthesis and is potentially the most rapid and efficient process for producing excess RNAs in stoichiometric amounts.

Global upregulation of gene expression can be directly determined using single-cell RNA sequencing, then normalizing such that the total number of cDNA fragments recovered is equalized between cells. This can be done for Pol II transcription using standard RNA-seq with oligo(dT) priming of 3’ polyadenylated transcripts, although that assumes proportionality between poly(A)+ and poly(A)- transcripts, which include small RNAs and long non-coding RNAs transcribed by Pol II but lacking a poly(A) tail. Total single-cell RNA sequencing is better, but since the vast majority of transcription in a cell is for components of the translational machinery, including ribosomal and transfer RNAs, this approach is not very precise. Rather, adding a spike-in to normalize bulk RNA-seq data is the most common approach for measuring hypertranscription [23]. Spike-ins can consist of adding a small number of heterologous cells, nuclei or purified DNA to each sample, where ideally the species should be sufficiently diverged so that there will be little if any cross-mapping to the other genome.

In some cases, single-cell RNA-seq with spike-ins is impractical, such as for many clinical samples, which are available as Formalin-Fixed Paraffin-Embedded (FFPE) or frozen sections, where total number of cells and degree of ploidy may be unknown, and where tumor purity typically varies. Standard practice is to perform bulk RNA-seq on the extracted tissue from archival tumor samples. However, detection of transcriptional amplification from RNA-seq data is complicated by the difficulty of directly determining the number of cells and their ploidy from polyadenylated or total RNA-seq data. To avoid this complication, two groups have reanalyzed bulk RNA-seq data and have modeled hypertranscription by counting recurrent mutations in target sites of known cancer drivers. In one approach, matched DNA sequencing and RNA sequencing datasets are deconvolved using tumor mutation signatures to separate tumor from normal and estimate the total amount of RNA per unit of DNA [17]. Transcript proportions were calculated for 6,590 bulk patient samples across 15 cancer types, revealing that high total mRNA expression correlates with increased risk of disease progression and death. In the case of early-stage lung cancer, total mRNA levels predicted reduced disease-free survival for 10 of 30 patients (*P* = 0.02) and by including % copy number alterations prediction accuracy improved to *P* = 0.003 [17].

In another approach, the authors took advantage of regions of loss of heterozygosity (LOH), in which germline and somatic hemizygous mutations are subject to selection [16]. LOH is common in cancer, and when there is no selection, the percentage of fragments with mutations in regions of LOH will be the same in the RNA as in the DNA. Hypertranscription in a tumor is revealed when this percentage is higher across regions of LOH in the RNA relative to the DNA, corrected for tumor purity calculated from the frequency of the normal allele within the same region. Using data from 7494 patient samples for 31 cancer types, hypertranscription was found to be widespread, with worse survival associated with higher levels of transcription. This study also identified 28 genes, including *MYC*, as putative drivers of hypertranscription. Importantly, hypertranscription confers a worse prognosis regardless of tumor type, ploidy or other predictive factors [16].

### Hypertranscription in cancer is driven by MYC and other regulatory factors

MYC was first shown to globally amplify transcription in cancer using spike-in normalization to quantify transcriptome-wide mRNA levels [20, 24]. MYC binding at active promoters increases the duration of transcriptional “bursts” [25], forcing positive supercoils, resulting in increased binding and engagement of topoisomerases [26, 27]. MYC upregulation may further amplify transcription by suppressing R-loop formation behind Pol II, thereby alleviating stalled elongation [28]. Thus, MYC not only promotes Pol II complex assembly and transcriptional initiation but also functions to attenuate the stress and DNA damage associated with amplified transcription. Although the effects of MYC and other oncogene TFs on the transcriptional apparatus are well-documented [29], molecular mechanisms have been debated [30]. One possibility is that oncogenic TFs shift transcriptional start site (TSS) preferences stochastically, increasing futile transcription on average, but ultimately resulting in selection for TSS shifts that facilitate tumor progression [31]. Moreover, high levels of MYC have been reported to saturate accessible canonical E-box TSS binding sites and “spill over” to lower affinity E-box sites at enhancers, in some cases acting cooperatively with other TFs (e.g. estrogen receptors) to promote neoplastic growth [32–34].

Although increased levels of MYC were commonly associated with hypertranscription, multiple ETS (E26 transformation-specific) family TFs, including ETS1, ERG and FLI1, have also been associated with hypertranscription [16]. Drivers of hypertranscription are not limited to TFs. For example, the Drosha endoribonuclease component of the small RNA Microprocessor complex [35] is the highest-ranking driver of hypertranscription, followed by HMGA1, a chromatin architectural protein that both displaces linker histone H1 and activates expression of the S-phase-dependent histone genes [36]. This latter observation suggests that one benefit of hypertranscription for a cancer cell is activation of S-phase histone gene transcription [37].

TFs do not act in a vacuum. Their activities are frequently downstream of signal transduction pathways and their functions are influenced by other TFs. MYC is a member of a gene regulatory network (GRN) of related basic-helix-loop-helix, E-box binding TFs connected by their dimerization with MAX or MLX. Members of the MYC network can promote or inhibit MYC’s normal and oncogenic activities [38], and perturbations of the network are associated with a wide range of cancers [39–42]. GRNs have evolved over ~1 billion generations of vertebrate evolution, and so it is hard to imagine how a cancer cell can “rewire” a GRN in a single cell generation. Yet some critics of traditional somatic mutation theory, in which cancer is driven by accumulation of random mutations, make exactly that argument, attributing the origin of cancer to epigenetic “attractors” [43] or to “plasticity” [44]. Hypertranscription provides a simple alternative to GRN rewiring or “epimutations” [45], as upregulation or amplification of *MYC* and members of its network is sufficient to increase precisely balanced biomolecular output in stem cells and cancer [18]. MYC also is required for endoreplication in Drosophila larval tissues [46] and polyploidy in the mouse placenta [47]. As expected for a driver of hypertranscription, MYC stimulates not only Pol II transcription, but also Pol I and Pol III transcription [48, 49]. Since cell growth and division is coupled to cellular production, hypertranscription acts as an invisible hand, analogous to Adam Smith’s metaphor for capitalist markets. In this case, hypertranscription supports and stimulates increased cellular proliferation. When constrained by developmental signaling cues cell proliferation maintains tissue homeostasis but when unbridled can lead to malignancy.

### Epigenomic profiling reveals hypertranscription directly in archival patient samples

Genomic profiling for cancer diagnostics has been rapidly advancing for liquid biopsies [50, 51], but much less so for solid tumors [52]. Profiling of surgical specimens is mostly performed on FFPE sections, which are routinely prepared from tissue samples for staining and microscopic examination by a pathologist. For more than a century, FFPEs have been accumulating in hospitals and clinics world-wide by the billions with rich metadata useful for retrospective studies [53]. Furthermore, rapid progress is being made in using computational tools to extract diagnostic information from these datasets [54]. However, damage caused by formalin fixation and RNA degradation during storage limits the value of RNA-seq, and mutations and epigenetic changes in regulatory elements that drive cancer are not generally detected.

DNA sequencing-based epigenomic profiling by ATAC-seq, CUT&Tag and related methods [55] has the potential of replacing WGS, panel sequencing and RNA-seq given the richness of gene regulatory information that is missed by these currently popular methods. For example, the ENCODE project has identified >2 million candidate cis-regulatory elements (cCREs) in the human genome, mostly sites of chromatin accessibility, which is two orders of magnitude more than the ~20,000 genes transcribed into 3’ poly(A) mRNAs that standard RNA-seq detects [56]. This large excess of accessible regulatory elements over genes is a universal feature of complex genomes, as a similar ~100-fold excess of cCREs over genes has been recently described for plants [57]. This dense genomic coverage makes possible extremely sensitive detection of hypertranscription by epigenomic profiling. Other features that can be sensitively detected are copy number variations, for example as seen for oncogene amplifications and clonal selection. Epigenomic profiling can also reveal differences at cCREs in cancer, as well as regulatory mutations, microRNA transcription, hypertranscription and histone gene transcription. Unlike RNA-based methods, degradation during long-term storage is not a practical issue with DNA-based epigenomics, where decades-old FFPE sections have been successfully profiled [37].

Both Assay for Transposase-Accessible Chromatin using sequencing (ATAC-seq) and Cleavage Under Targets and Tagmentation (CUT&Tag) have been successfully applied to FFPEs [58, 59], although most success has been reported for Cleavage Under Targeted Accessible Chromatin (CUTAC), which uses low-salt tagmentation to profile epitopes at sites of open chromatin [60]. When applied to standard FFPE pathology sections using an antibody to the Serine−5 CTD-phosphorylated (Ser5p) paused form of Pol II, FFPE-CUTAC profiles active regulatory elements genome-wide, including promoters and enhancers [60]. Pol II-Ser5p FFPE-CUTAC takes advantage of the absence of lysines in the 52 tandem copies of YSPTSPS that comprise the CTD. This maximizes the contrast between sites of paused Pol II and the background of lysine-rich histones that are heavily cross-linked by formalin fixation. For example, in mouse brain tumors, Pol II-Ser5p FFPE-CUTAC identified active regulatory elements for down-regulated tumor suppressor microRNA genes not detected using standard RNA-seq. Pol II-Ser5p FFPE-CUTAC also uncovered hypertranscription and HER2 amplifications in human cancer samples (Fig. 2B), and in the case of a breast tumor, detected clonal selections of nearby CDK12 and MED1 genes, both known to predict poor prognosis in HER2-driven breast tumors [37]. Pol II CUTAC epigenomic profiles can be integrated with RNA-seq data from existing public archival data for diagnostics [37] using powerful machine learning approaches [61]. However, tumor samples are heterogenous, and bulk analysis obscures this cell-type diversity. Although single-cell profiling methods suffice for liquid tumors, spatial profiling is required to delineate tumor from host tissue. Tumor cells also manipulate their setting to attenuate host defenses and to condition their microenvironment for their own metabolic needs, further complicating molecular analysis.

Both spatial transcriptomics and spatial chromatin profiling have been used to identify cell types and gene expression patterns in the cores of solid tumors, at their margins, and at the margins of host tissue. But two recent studies show that chromatin-based assays can provide additional genomic information describing the histories of tumors and perhaps their origins [62, 63]. Accessibility mapping by ATAC-seq also tagments the nucleosome-free mitochondrial genomes, and the relatively high mutation rate of the mitochondrial genome provides new SNPs within samples that can be used to reconstruct cell lineages within solid tumors, informing the trajectory of cancer progression within that tumor. Similarly, spatial profiling has been used to infer changes in copy number variations, which can be used for lineage reconstruction and some of which are expected to be associated with oncogenic drivers [64]. Although spatial profiling of tumor samples is too specialized and expensive for routine laboratory or diagnostic applications, the potential of spatial transcriptomic and chromatin profiling has motivated the development of inherently affordable platforms [65].

### RNA Polymerase II at histone genes predicts outcome in human cancer

**Fig. 3:**
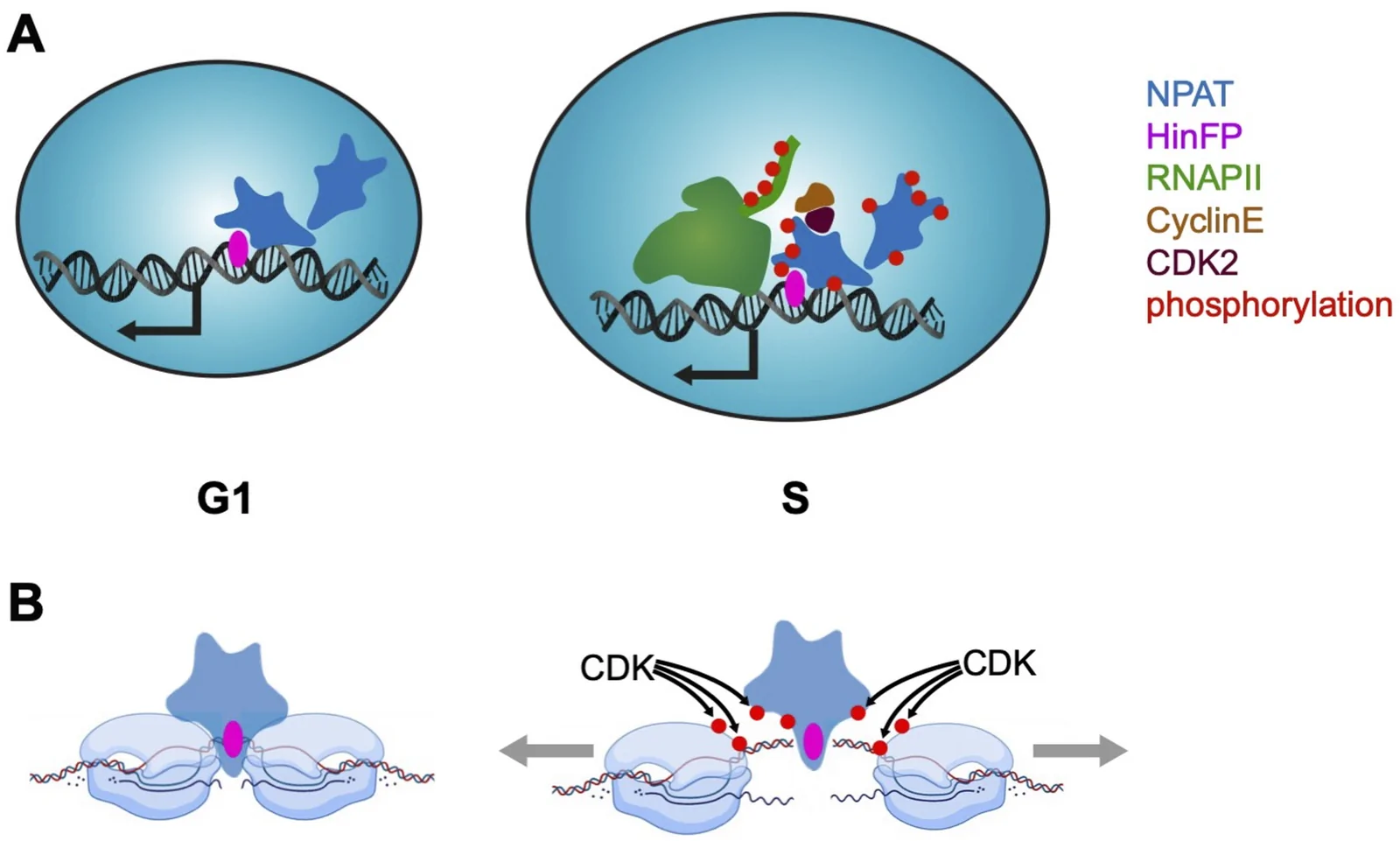
The Histone Locus Body (HLB). **A** NPAT is a major structural protein of the Histone Locus Body that serves as a transcriptional activator targeted by Cyclin E/CDK2 at the G1-to-S transition. The HLB (blue sphere) encompasses histone genes throughout the cell cycle. The scaffolding protein NPAT is constitutively localized to the HLB through interaction with the HinFP TF that binds histone gene promoters. As cells transit into S phase, cyclinE/CDK2 and Pol II localize to the HLB and the amount of NPAT increases. Phosphorylation of NPAT and Poll II activates histone gene transcription. **B** Protein interactions within the HLB may be modulated by phosphorylation. We propose that at bidirectional histone gene promoters, weak interactions between NPAT IDRs and the unstructured Pol II CTD hold transcriptional machinery in place (left). At the G1/S transition, dense phosphorylation of NPAT by CDK2 and of the CTD by CDK7 cause electrostatic repulsion between them, propelling Pol II downstream for rapid activation (right)

Nucleosomes are defining features of eukaryotic cells, and in animals, genes for the canonical histone proteins that form the nucleosome core (H2A, H2B, H3 and H4) and linker (H1) are produced within distinct nuclear condensates, called Histone Locus Bodies (HLBs) (Fig. 3A). In human cells, most histone gene transcription occurs in the HLB that forms at the major cluster on Chromosome 6p22.1 and in the HLB at the minor cluster on Chromosome 1q21.1 [66]. With about a dozen each of the five canonical histones, not all of which are fully active during S-phase, an extraordinarily high level of Pol II transcription is required during S-phase to produce enough histone protein to package the full replicated genome every cell cycle, estimated to be nearly 5% of a cell’s total protein [67]. Considering that there are ~10 [16] cell cycles in a human lifetime, histone gene transcription [68], and subunit stoichiometry [69, 70] must be tightly regulated to be compatible with cellular life.

Canonical histone mRNAs in humans are neither spliced nor polyadenylated. Rather an RNA 3’-end stem-loop is bound by Stem-Loop Binding Protein (SLBP), which directs the HLB-specific U7 snRNP 3’-end processing machinery, an ancient mechanism that resembles the prokaryotic mRNA 3’ stem-loop bound by NusA [71, 72]. As a result, mRNAs that encode canonical histones are nearly absent from standard RNA-seq datasets that use oligo(dT) priming of the 3’ poly(A) tail. The overwhelming reliance on RNA-seq to profile gene expression in cancer is perhaps one reason why hypertranscription and histone gene transcription have been overlooked in discussions of cancer epigenetics [6, 43, 44].

In contrast to RNA-seq, methods that detect and quantify Pol II can reveal the high abundance of active transcription within the HLB. For example, in Drosophila early embryos undergoing rapid syncytial cell cycles, HLBs stand out as the largest Pol II-enriched transcriptional condensates at S-phase [73], and become even more enriched at the expense of other Pol II condensates when zygotic genome activation is prevented [74]. Moreover, mouse S-phase embryonic stem cells could be identified exclusively based on the high levels of Pol II at histone genes, with a 40% genome-wide reduction during the transition from G1 to S phase [75]. A dedicated structural component of the mammalian HLB, NPAT, is also the major transcriptional activator for S-phase-dependent histone gene regulation [68]. NPAT precisely co-localizes with Pol II at the human S-phase-dependent histone gene promoters, and at bidirectional promoters, co-localization spans the ~150-bp at the 5’ ends of both genes [76] (Fig. 3B, left). We propose that the master regulator of the G1-to-S transition, Cyclin E/CDK2, phosphorylates NPAT, and together with Serine−5 phosphorylation of Pol II CTDs [37], the repulsion of so many phosphates overcomes the weak interactions holding these two proteins together, releasing Pol II downstream for productive elongation (Fig. 3B, right). These cytological and epigenomic studies suggest that histone genes are “greedy” for Pol II at S-phase and support the possibility that histone synthesis is rate-limiting for S-phase progression. In this way, hypertranscription may benefit a cancer cell in large part by overproducing histones to hasten the G1 to S phase transition [77] and proliferate faster than neighboring cells [37].

Interestingly, MYC becomes highly enriched at the Drosophila HLB across cell types exclusively during S-phase [78], and overexpression of MYC in Drosophila wing imaginal discs and in mammalian cell lines shortens the transition from G1 to S phase [79, 80]. These observations suggest that the same mechanism that has evolved for upregulation of histone gene transcription during replication also drives hypertranscription in cancer. If indeed histone gene overexpression during S-phase is a cancer driver, then it may be possible to predict outcome based on histone expression levels alone. In a test of whether histone gene overexpression could be used to predict outcome in cancer, Pol II Ser5p FFPE-CUTAC was found to significantly correlate with WHO grade when applied to 30 meningiomas, intracranial tumors that are usually benign, but can recur and be fatal [37]. By intersecting Pol II FFPE-CUTAC signal within gene regions with RNA-seq data from 1298 meningioma patient tumor samples mostly with known outcomes, the 5 tumors with high Ser5p signals were predicted to be recurrent and the other 25 benign. These 5 meningiomas also showed an excess of whole-arm copy number changes relative to the 25 predicted benign, with losses more frequent than gains, a level of accuracy that is expected to occur by chance at 1 × 10^–8^, performance that was much better than that obtained using other cancer biomarkers. This association between the overall level of Pol II at histone genes and malignancy suggests that histone gene overexpression might also be a biomarker of chronological age, the most important predictor of cancer incidence [81].

### Hypertranscription and replicative aging

MYC both drives hypertranscription and suppresses replicative senescence, which is permanent arrest of cell proliferation, common in aging tissues [82, 83], consistent with the possibility that hypertranscription underlies epigenetic clocks. Biological clocks based on DNA methylation changes have been the default epigenetic predictors of chronological age for over a decade [84]. However, correlations between age and methylation of specific CpG dinucleotides can vary substantially both between tissues and between individuals and are sensitive to environmental factors [85]. As a result of these and other confounding variables that complicate the use of DNA methylation changes for estimating chronological age [86], recent efforts are aimed at improving aging biomarkers and analysis methods [87]. Epigenetic clocks based on other biomarkers have been proposed, often using chromatin markers that are linked to Pol II activity [88]. For example, aging in the mouse liver is associated with decreased Pol II promoter-proximal pausing and increased promoter and enhancer accessibility [89]. Also, total long-read RNA-seq suggested that Pol II elongation speed increases with age in animals [90], although another study suggested decreases in elongation speed with age for mouse brain, liver and kidney [91]. Dysregulation of Polycomb gene silencing with age was seen both in mammalian cells [88] and in the Drosophila gut epithelium, where global Pol II abundance decreased only late in life [92]. Considering that the incidence of cancer increases with age, it seems likely that hypertranscription can serve as both a positive biomarker of cancer and a positive or negative biomarker of chronological age [81].

Histone gene transcription has also been implicated in the aging process. For example, an age predictor based on human ENCODE Chromatin Immunoprecipitation with sequencing (ChIP-seq) data for 7 histone modifications identified histone genes as one of the best predictors of age, where active modifications correlated with youth and silencing with old age [86]. Consistent with these observations, elevation of histone gene expression extends yeast lifespan [93], and dramatic elevation of Pol II at core histone genes with a global decrease over other Pol II genes is a normal process in the gut of aging Drosophila adults [92], organisms that completely lack DNA methylation. In the fly gut, histone gene overexpression may drive stem cell overproliferation that occurs as differentiated enterocytes decrease in number with age [92]. Overexpression of histones H3 or H4 in Drosophila decelerates Pol II in aging flies and compensates for histone loss, reducing senescence and increasing lifespan [90]. Similarly, in a mouse model of aging, expression of S-phase-dependent histone genes decreased with age but could be reversed by Oct4, Sox2 and Klf4 Yamanaka factors that drive cellular rejuvenation [94]. Together, these observations suggest that reduced histone production is a causal factor driving senescence in aging tissues, but that cellular aging can be reversed by restoring histone production [92].

### Centromere breaks initiate chromosomal instability in cancer

Although the extent to which histone overexpression is a general biomarker for malignancy and chronological age remains to be determined, whole-arm aneuploidy is a well-documented hallmark of cancer. Aneuploidy in cancer was first described in 1890 [3], and the chromosomal instability resulting from aneuploidy is also recognized as a cancer hallmark [1]. Initiation of aneuploidy is a very early event in cancer, as very low levels of aneuploidy are consistently detected in normal breast tissue by single-cell DNA sequencing [95]. Most aneuploidy is caused by breaks at centromeres or pericentric regions of the chromosome. For example, a pan-cancer study of public whole-genome sequencing (WGS) data revealed that 86.5% of breakpoints within copy number variants corresponded to whole-arm gains, losses or rearrangements [96]. Examination of both RNA-seq and WGS data revealed that simply counting the number of whole-arm gains and losses predicted outcome in cancer [97]. Whole-arm losses predominate over gains, a finding that is counter-intuitive, insofar as 1/500 newborns carry a trisomy but no newborn with a monosomy is known to have come to term [98]. These surprising observations challenge the general assumption that initial aneuploidies in cancer follow from errors in chromosome segregation [99–101], but rather suggest that they result from centromere breaks followed by positive selection for whole-arm loss or gain.

High levels of whole-arm aneuploidies correlate with high levels of Pol II at histone genes in diverse cancers [37]. Consistent with this observation, Gene Set Enrichment Analysis (GSEA) based on pan-cancer RNA-seq data revealed a strong positive association between aneuploidy and histone genes after applying a linear mixed model (LMM) to remove confounding factors such as tumor purity and gene co-regulation [102]. GSEA-based association of histone genes with aneuploidy improved from weaker to much stronger than associations observed for other cell-cycle-dependent gene sets, such as those for cell cycle checkpoints and mitosis. For example, the ~10-fold stronger GSEA association of aneuploidy with histone gene expression than with expression of DNA replication genes implies that a root cause of chromosomal instability in cancer is the requirement for histone packaging to prevent DNA damage behind the replication fork [101].

While most cancer types show an excess of whole-arm aneuploids resulting from centromere breaks in cancer, most glioblastomas are characterized by gain of whole Chromosome 7 and loss of whole Chromosome 10, with relatively low levels of whole-arm gains or losses [97, 103]. However, glioblastoma, the most invasive of all brain tumors, also stands out as showing the highest association between aneuploidy and histone gene expression by far in LMM-filtered GSEA [102], which suggests a need for high levels of histones for glioblastoma to thrive. This observation is consistent with centromere breaks occurring as very early events in tumor development, as glioblastomas proliferate rapidly only during late stages. Glioblastomas provide an illuminating exception to the more general observation that high levels of Pol II at histone genes correlate with the number of centromere breaks [97].

**Fig. 4:**
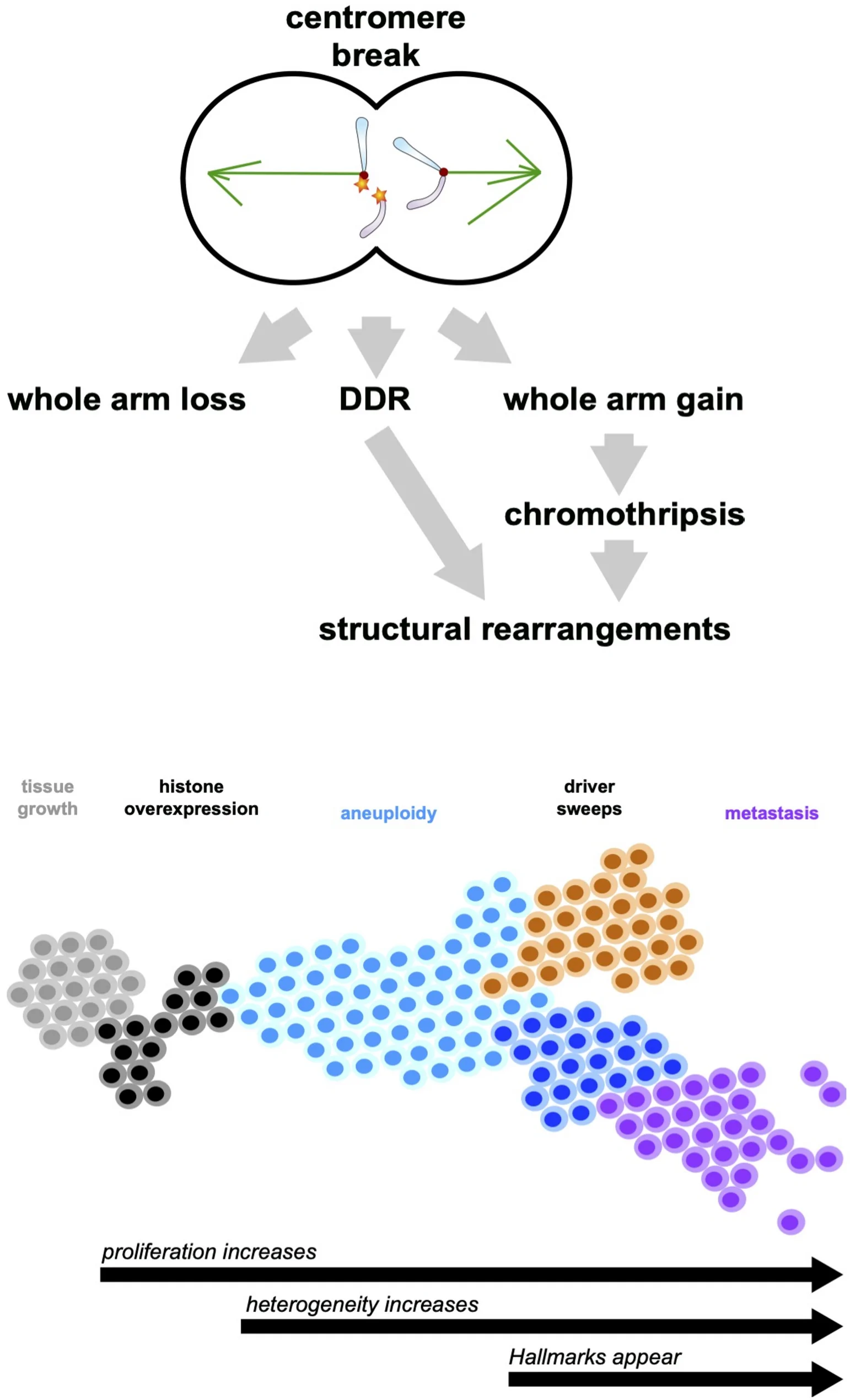
A model for cancer progression from a single cell: Histone overexpression causes centromere breaks, whole-arm gains and losses, and micronuclei. **A** Histone H3|H4 dimers are deposited along chromosome arms behind the replication fork, and CENPA|H4 dimers are deposited at centromeres and maintain centromere identity. In cancer cells, hypertranscription drives production of excess H3|H4 dimers, which compete with CENPA|H4 for centromere assembly [105]. Loss of CENP-A causes replication-transcription conflicts, causing centromeric DNA breaks [106]. Segregation of broken chromosomes can result in whole-arm gains or losses, or a free arm is encapsulated in a micronucleus, resulting in DNA damage and release of extrachromosomal DNA circles [110]. Grey shading: euploid nucleus; blue shading: aneuploid nucleus; red shading: micronucleus. **B** MYC overexpression results in histone overexpression, leading to centromere breaks and aneuploidy early in cancer, and mitotic mis-segregation result in increasing heterogeneity. Amplifications and clonal selection results in selective sweeps of driver gene copies which accompany the cytologically observable cancer hallmarks, with increasing probability of metastasis as cell numbers and heterogeneity increase

How can overexpression of S-phase-dependent histone genes cause centromere breaks? An important clue comes from studies of CENP-A, a histone H3 variant that replaces H3 in centromeric nucleosomes in nearly all eukaryotes. In mammals, centromeres are epigenetically defined by the presence of CENP-A, in that neocentromeres that form fully functional kinetochores can arise at sequences that are unrelated to centromeric satellite DNA tandem repeats [104]. In a chromatin fiber assay, overexpression of H3 was shown to deplete CENP-A in both Drosophila and human cells [105], and depletion of CENP-A using RNAi in human cells resulted in centromere breaks at S-phase, evidently resulting from replication-transcription conflicts [106] (Fig. 4A). CENP-A deposition requires Pol II transcription through centromeres during G1 of the cell cycle [107], and excess H3/H4 dimers competing with CENP-A/H4 dimers might leave centromeric regions relatively unprotected when the replication fork passes through.

Human centromeres are characterized by a bipartite structure, and breaks between the halves result in both lagging chromosomes and merotelic attachments that can result in segregation of arms to opposite poles [108]. During subsequent mitoses, arms may be lost or gained, and lagging chromosomes that are not included in the nucleus at telophase may form micronuclei [109]. It is thought that the high surface area to volume ratio of micronuclei can lead to loss of factors that protect genomic DNA from damage, eventually resulting in extrachromosomal DNA circles that can arise from transcriptional hot spots and are characteristic of cancer cells [110]. Thus, one outcome of hypertranscription, histone overexpression, may underlie not only cancer aggressiveness at early stages, but also genomic changes responsible for much of the heterogeneity that is observed during invasion and metastasis (Fig. 4B).

### Hypertranscription drives microRNA production during cancer progression

Another outcome of hypertranscription is overproduction of microRNAs, ~22-nucleotide RNAs involved in post-transcriptional mRNA silencing, such as those normally produced in high abundance from multi-copy ribosomal gene arrays [111]. microRNAs, together with small nucleolar RNAs and long non-coding RNAs, are exported in extracellular vesicles to condition a cell’s environment and help resist immune invasion [112]. microRNAs are exceptionally sensitive circulating cancer biomarkers, and those produced by the ribosomal DNA spacer promoter [111] are the most significantly upregulated in multiple human cancers [113, 114]. Hypertranscription-associated upregulation of regulatory RNAs and genetic processes that result in tumor heterogeneity, such as chromothripsis [115] and generation of extrachromosomal DNA circles [110], obscure the earliest events in cancer; however, studies of histone gene regulation in model systems and human tumors have begun to reveal underlying mechanisms.

microRNAs regulate gene expression by base-pairing with the 3’ untranslated regions of mRNAs in the cytoplasm, targeting them for degradation and silencing [116]. As a microRNA “seed” comprises only 6–8 base pairs, the number of potential targets is vast, with effectiveness increasing in proportion to the microRNA:mRNA stoichiometry. Studies of microRNAs in *C. elegans* have revealed the complexity of gene regulation by microRNAs that target TF mRNAs, as reducing their levels below a critical threshold for expression of some genes may not be enough for other genes, causing imbalances that are likely to be detrimental and selected against [117]. However, microRNAs can provide stability to GRNs [118], for example when a TF activates the gene for a microRNA that targets a different TF, which then activates a second microRNA gene that targets the first TF [119]. Such GRNs might not be able to tolerate more than minor genetic or epigenetic disruptions, but would likely tolerate low levels of hypertranscription, which maintains the balance of GRN components.

Although microRNA targets can be predicted computationally, the combination of low sequence specificity and concentration dependence of TFs for their binding sites severely complicates determination of their specific roles within GRNs. Therefore, it is reasonable to expect that the most abundant microRNAs in a cell type are those most likely to be functionally important and so mobilized by cancer cells for targeting TF mRNAs. This raises the possibility that hypertranscription creates opportunities for individual cancer cells to take advantage of abundant microRNAs that target tumor suppressor TF mRNAs [117]. For example, two microRNAs that are abundantly produced from the ribosomal RNA gene arrays and exported from cells in exosomes [37] are also the most upregulated of all circulating microRNAs from plasma or urine from cancer patients [120, 121]. These promiscuous increases in expression of the same gene are difficult to account for by targeted upregulation, but are as expected for hypertranscription, where the most abundant microRNAs are also those that will be the most upregulated. That is, hypertranscription can be likened to the rising tide that lifts all boats so that tallest mast will still be the tallest at high tide.

### Negative feedback by histone H4 regulates S-phase-dependent histone gene expression

In mammalian cells, the mostly unstructured >1400 amino acid NPAT protein is the major structural component of the HLB and a target of phosphorylation by Cyclin E/CDK2, the kinase that initiates S-phase [68]. In mouse models of cancer, activation of CDK2 phosphorylation at the G1-to-S boundary is rate-limiting for cell-cycle duration, independent of other cancer hallmarks [77]. Together with the requirement for high-level S-phase-specific histone production to package DNA behind the replication fork [75], these observations suggest that activation of histone gene transcription drives cancer initiation through phosphorylation of NPAT and other CDK2 targets.

Much of our understanding of the HLB comes from work in Drosophila, where the S-phase-dependent histone genes are present within >100 tandem copies of an ~5-kb unit encoding all 5 histone subunits [66] within a heterochromatic region of the genome [122]. Replacement of the entire array with an engineered multicopy transgenic array has made it possible to perform precise genetic perturbations in whole organisms [123], not feasible in mammalian models where the individual histone genes are scattered within multiple clusters [124]. HLBs are readily identified as nuclear puncta by fluorescence microscopy, allowing for a cytological assay to be performed in flies expressing Dendra2-tagged histones [125]. This assay was applied to Drosophila testes, in which rapidly cycling cells occupy the apical tip and exit the cell cycle at G2 to form gonial cells. Knockdown of essential genes required for epigenetic silencing showed no effect on histone expression, suggesting a novel form of cell-cycle-dependent gene silencing [126]. Knockdown of individual genes for canonical or variant histones likewise had no effect, with the striking exception of canonical Histone H4, implying negative feedback regulation of the HLB by H4. Indeed, H4 was observed to co-localize with NPAT in HLB puncta not only in Drosophila, but also in human cells, where CUT&Tag profiling also showed precise H4 co-localization with NPAT. Together, these cytological and epigenomic observations imply direct negative feedback regulation of the HLB by Histone H4, coinciding with the silencing of histone gene expression outside of S-phase. Non-nucleosomal histones have been proposed to act as chromatin regulators in other contexts [68, 125], and virus-encoded H4 has been “weaponized” by braconid parasitic wasps to silence host H4 expression in moth larvae prey, resulting in viral H4-containing nucleosomes that favor wasp larval development [127].

Direct negative feedback regulation of the HLB by H4 binding to NPAT leaves open the question of how repression is relieved to allow Cyclin E/CDK2 phosphorylation of NPAT and activation of histone gene transcription. Recently, an elegant solution to this problem was revealed with the demonstration that the PRMT5 arginine symmetrical dimethyltransferase fulfills that role: Among PRMT5’s multiple histone targets is the arginine−3 residue of histone H4 (H4R3), and in human cells, inhibition of PRMT5 causes downregulation of both histone mRNAs and histone proteins within 15 min of drug treatment [128]. Based on phylogeny, PRMT5 appears to be as ancient as the eukaryotes [129], which suggests that the H4/PRMT5 feedback cycle is an ancestral mechanism for S-phase-specific regulation of histone gene transcription by Pol II.

### Hypertranscription may be targeted by widely-used cancer therapies

Unlike targeted therapies, RNA polymerase inhibitors and degraders are potentially effective against many or all cancer types, limited more by pharmacokinetic properties, toxicity and efficacy [130]. Some small molecule compounds target “transcriptional addiction” [29] by inhibiting kinases that phosphorylate Serine−2 of the Pol II CTD (YSPTSPS)_52_ repeat or by targeting MYC [131]. These include CDK9, which is a component of the positive transcription elongation factor b (P-TEFb) complex, necessary for pause release [132], and CDK12 and CDK13 that promote elongation downstream [133]. The discovery that over 100 kinases phosphorylate Tyr-1, Ser-2, Thr-4, Ser-5 or Ser-7 of the Pol II CTD in vitro suggests that many other kinase inhibitors and degraders act directly on Pol II and raise questions about their presumed physiologically important target proteins [12].

Hypertranscription in cancer might also be the physiological target of classes of DNA-intercalating drugs that are widely used in standard chemotherapy [9]. Among these are the anthracyclines and curaxins, which have been shown to cause non-covalent chromatin damage, in some cases without detectable damage to DNA. For example, the planar moiety of the anthracycline aclarubicin intercalates between DNA base pairs and inhibits topoisomerases, with torsional effects that increase Ser-2 phosphorylation of the Pol II CTD, likely a result of retarding Pol II elongation, thus increasing the duration of exposure to transcriptional kinases [134, 135]. As anthracyclines are given annually to ~1 million cancer patients in the US alone, a better understanding of how they might counteract hypertranscription may have a major impact on the development of improved chemotherapeutic compounds and treatment regimens.

The realization that PRMT5 dimethylation of H4R3 drives histone gene transcription has immediate implications not only for understanding how hypertranscription drives cancer, but also for developing new pan-cancer therapeutics. High PRMT5 expression has a poor prognosis in multiple tumor types, and PRMT5 has been identified as a cancer therapeutic target for more than a decade [136, 137]. Currently, small molecule inhibitors and degraders of PRMT5 are perhaps the most promising anti-cancer drugs in the epigenetics field [138], with strong evidence of effectiveness [139]. Although PRMT5 regulates other cellular processes, such as splicing and mRNA escape from chromatin [140, 141], it is likely that PRMT5’s ability to directly derepress HLB transcription at the G1-to-S boundary accounts for the anti-cancer efficacy of PRMT5 inhibition. Consistent with a critical role in driving events at the replication fork, PRMT5 inhibitors synergize with inhibitors of the DNA damage response [142], in support of our speculation that histone overexpression drives early events in cancer [143]. Histone overexpression as a cancer driver fulfills predictions of atavistic theory, that cancer cells “are heirs to an ancient toolkit and a basic mode of survival” [144].

**Fig. 5:**
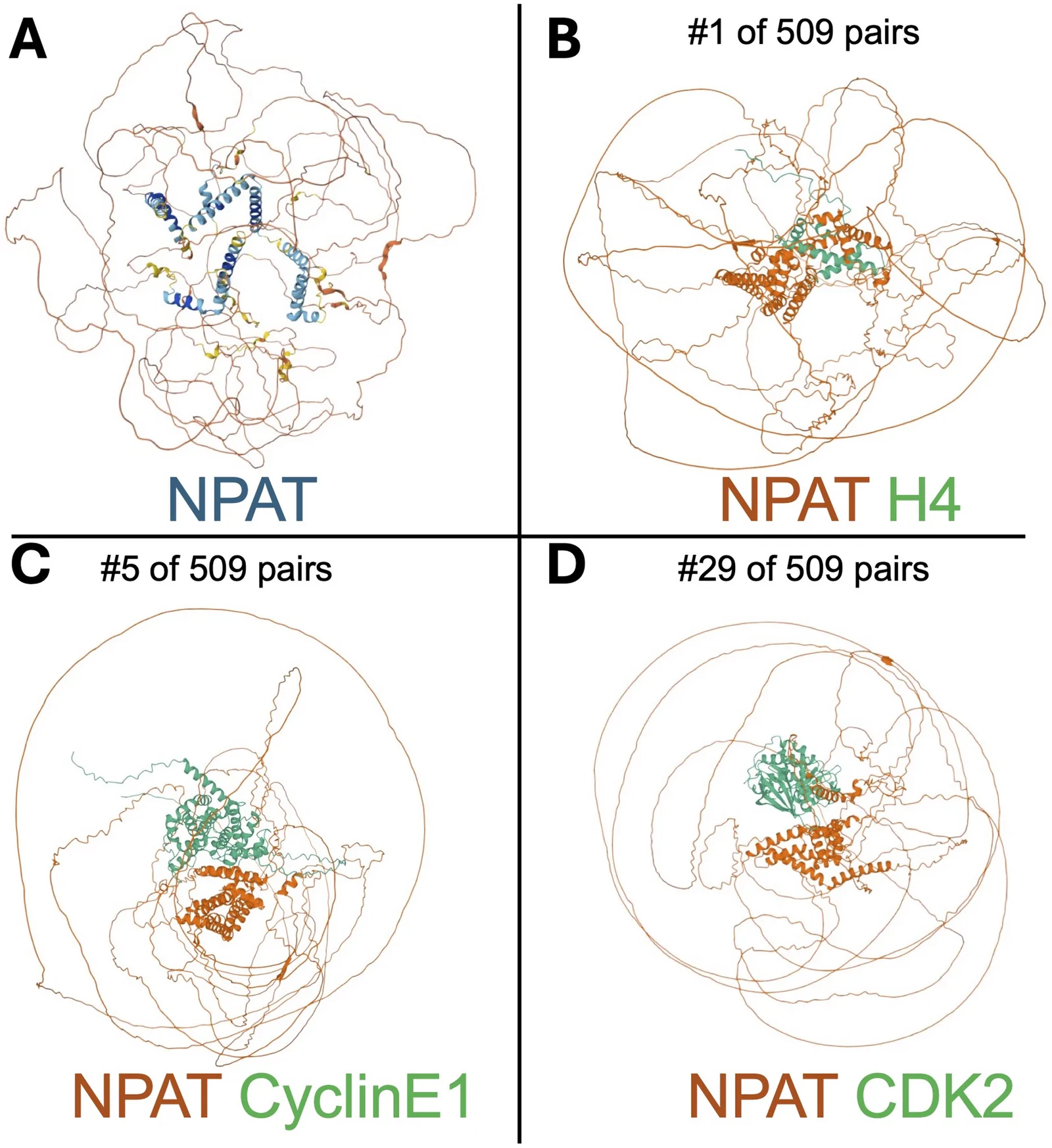
AlphaFold Multimer predicts condensation of NPAT alpha helices with partner binding. Of the 509 proteins predicted by AlphaFold Multimer to interact with NPAT (**A**), the top hit is Histone H4 (**B**), showing condensation with intersecting alpha-helices. The fifth best hit is CyclinE1 (**C**) and its partner kinase, CDK2, is 29th on the list (**D**), in both cases showing condensation of NPAT alpha helices. Other highly ranked proteins include known NPAT interactors such as HinFP (#9), LSM10 (#25) and SYMPK (#35), which suggests that the HLB repressor H4 and the Cyclin E1/CDK2 activator of S-phase-specific histone transcription are stably bound within the HLB

What is the molecular mechanism of repression and derepression of histone gene transcription? Intriguing clues come from recent advances in protein–protein interaction prediction based on AlphaFold Multimer [145]. Human NPAT is predicted to be almost completely unstructured based on AlphaFold [146] and lacks a DNA-binding domain (Fig. 5A). This suggests that weak interactions [147] with the unstructured Pol II CTD trap NPAT on the DNA, consistent with the observation that highly specific NPAT binding at the clustered histone genes is resistant to high salt washes used for CUT&Tag [148]. NPAT has 509 predicted interaction partners modeled to fold together with other unfolded regions, condensing into a putative alpha helical-rich complex [146]. The most probable interaction partner is Histone H4, with Cyclin E1 ranked #5, and its partner, CDK2, ranked #29 (Fig. 5B–D). Although caution is warranted in interpreting AlphaFold Multimer intermolecular interactions, prediction accuracy is especially high for intrinsically disordered regions, with a 76% success rate [149].

## Conclusion

The field of cancer epigenetics has evolved as DNA and chromatin profiling technologies have advanced. Results from bisulfite DNA sequencing and hybridization microarrays during the 1980s and 1990s led to the realization that global DNA hypomethylation coupled with promoter methylation are general features of the cancer epigenome [5]. During the next two decades, Chromatin Immunoprecipitation-based methods led to the discovery of protein-based genomic features resulting from the sequence-specific action of oncogene and tumor suppressor transcription factors [150]. These include histone modifications, their “writer” enzymes and “reader” proteins, histone variants, ATP-dependent chromatin remodelers, and silencing complexes [151]. Over the past decade, the use of spike-ins and advances in single-cell RNA sequencing has led to the realization that transcriptional amplification driven by MYC and other positive regulators of transcription is widespread in cancer [16]. The common denominator across cancer types is hypertranscription, and yet curiously, the transcriptional machineries, targets of some of the most effective anticancer drugs, are never mentioned in discussions of cancer epigenetics [6, 43–45] or hallmarks [1, 7]. The very recent availability of practical methods for profiling RNA polymerases in patient samples promises to provide new opportunities for understanding and diagnosing cancer and for the development of improved therapeutics.

## Data Availability

No datasets were generated or analysed during the current study.
